# Supplementary material for: Identification of photoperiod-induced specific miRNAs in the adrenal glands of Sunite sheep (Ovis aries)
Source: Front Vet Sci. 2022 Jul 22;9:888207. doi: 10.3389/fvets.2022.888207 (PMC9354845; doi:10.3389/fvets.2022.888207)
Supplement: Supplementary file 1 [file Data_Sheet_1.ZIP › Supplementary file/miRDeep2 file.zip!/miRDeep2 file/Know_Structure/SP42A2/oar-mir-103.pdf]

miRBase precursor : oar-mir-103
Total read count : 21254
oar-miR-103 read count : 21253
remaining reads : 1

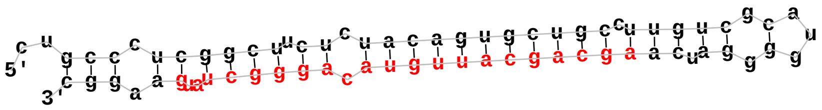

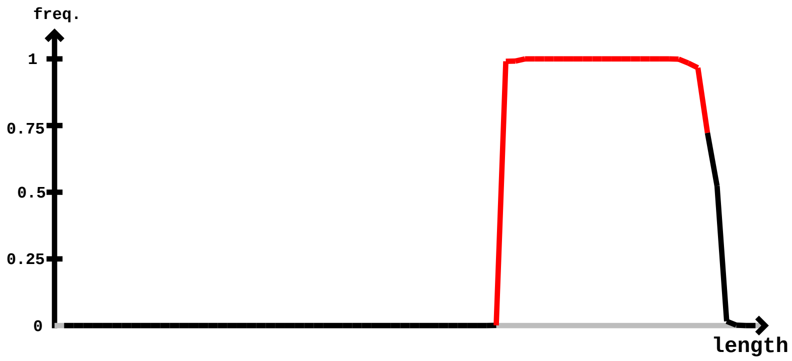


```
                                                oar-miR-103
5'- cugcccucggcuucucuacagugcugccuugucgcauggggaucaagcagcauuguacagggcuaugaaggc   -3'    exp
    ..(((.((((((.((.(((((((((((.(((((.(...).)).))))))))))))))).))))))..)).)))   reads     mm         sample
    ........ggcuucucuacagugcugccuugu........................................   1         0          seq
    ............................................aagcagcauuguacagggcuaug.....   5         0          seq
    ............................................aagcagcauuguacagggcuauga....   12        0          seq
    .............................................agcagcauuguacagggc.........   21        0          seq
    .............................................agcagcauuguacagggcu........   311       0          seq
    .............................................agcagcauuguacagggcua.......   374       0          seq
    .............................................agcagcauuguacagggcuau......   5176      0          seq
    .............................................agcagcauuguacagggcuaug.....   4200      0          seq
    .............................................agcagcauuguacagggcuauga....   10633     0          seq
    .............................................agcagcauuguacagggcuaugaa...   293       0          seq
    .............................................agcagcauuguacagggcuaugaaA..   32        1          seq
    .............................................agcagcauuguacagggcuaugaag..   3         0          seq
    ..............................................gcagcauuguacagggcuau......   4         0          seq
    ..............................................gcagcauuguacagggcuaug.....   6         0          seq
    ..............................................gcagcauuguacagggcuauga....   10        0          seq
    ................................................cagcauuguacagggcu........   1         0          seq
    ................................................cagcauuguacagggcua.......   2         0          seq
    ................................................cagcauuguacagggcuau......   8         0          seq
    ................................................cagcauuguacagggcuaug.....   22        0          seq
    ................................................cagcauuguacagggcuauga....   124       0          seq
    ................................................cagcauuguacagggcuaugaa...   14        0          seq
    .................................................agcauuguacagggcuaug.....   1         0          seq
    .................................................agcauuguacagggcuauga....   1         0          seq
```
